# Cardiac digital twins: Modelling the arrhythmic substrate of chemotherapy

**DOI:** 10.1113/JP290313

**Published:** 2026-01-29

**Authors:** Radomir Chabiniok, Vlad G. Zaha

**Affiliations:** ^1^ Heart Center, Children’s Health, Division of Cardiology, Department of Pediatrics UT Southwestern Medical Center Dallas Texas USA; ^2^ Division of Cardiology, Department of Internal Medicine UT Southwestern Medical Center Dallas Texas USA

**Keywords:** cardio‐oncology, *in silico* medicine

Anthracyclines, such as doxorubicine (DOX), are a ‘double‐edged sword’, very effective as anti‐cancer agents but with unpredictable cardiovascular toxicity in 5–10% of patients. Their toxicity can manifest acutely due to myocellular injury, or a long time after treatment due to cumulative cellular damage and impaired repair mechanisms. DOX can lead to microvascular and myocardial dysfunction, reactive fibrosis, impaired systolic and diastolic function, and progression to heart failure, and can also increase the vulnerability to ventricular arrhythmias (VAs). All these cardiotoxic effects impact negatively long‐term outcomes in cancer survivors. Objective monitoring of cardiotoxic effects is essential for early detection, assessment of disease stage and response to therapy to improve long‐term outcomes.

Echocardiography is the most prevalent cardiac imaging modality. However, limited acoustic windows and variability in technical acquisition performance decrease the sensitivity of this method, especially for early mechanical changes. In contrast, access to cardiac magnetic resonance imaging (MRI) is more limited than to echocardiography, but cardiac MRI is the gold standard for measurement of cardiac morphology and function, providing measurements with a high level of accuracy. Nevertheless, even the best current clinical methods taken in isolation have a limited predictive ability for negative outcomes (Lyon et al., 2022).

The work by Villar‐Valero et al. (2026) aimed to predict the risk of VAs after exposure to DOX. The authors created a digital twin (Corral‐Acero et al., 2020), that is an *in silico* model based on physical and physiological assumptions, that integrates data acquired *in vivo*. Digital twins allow clinical data to be explored in unprecedented ways, providing new informational dimensions, and can be particularly helpful when analysing multi‐modality data. The biophysical characteristics of the model lend themselves to predictivity, allowing them to assess risks of adverse events (e.g. VAs), or *in silico* testing of therapeutic scenarios (Gusseva et al., 2025; Sermesant et al., 2012). Whilst digital twins have great potential for precision medicine, there is still a substantial gap between the modelling methods and their successful clinical implementation.

For clinical translations, the digital twins need to be personalized using clinical data and provide the output in clinically acceptable time. Therefore, an optimal balance between model complexity and efficient processing is essential. In Villar‐Valero et al. (2026), the authors employed the phenomenological model of Mitchell–Schaffer (MS) for transmembrane potential generation, and a reaction–diffusion model (monodomain model) for propagation in the tissue, for which they implemented a Lattice–Boltzmann method (LBM) solver. Whilst the MS model does not consider the full biophysical complexity of the cellular membrane, it is easier to personalize using clinical data compared to calibrating several ionic channels in comprehensive biophysical models. The LBM, belonging to more recent numerical methods, allows the direct use of regions segmented from imaging data and therefore avoids the challenging step of creating a computational mesh used in the finite element method (FEM). In addition, the LBM is highly parallelizable and suitable for the implementation on general‐purpose graphical processing units (GPUs), which can be accessible in clinical settings. The computations therefore do not need to be outsourced from the clinical department. Villar‐Valero et al. (2026) validated their LBM implementation against the modelling benchmark by Niederer et al. (2011): they demonstrated an excellent agreement in the wavefront propagation and revealed a consistent 140‐fold speedup in computational time compared to the FEM. Overall, the choice of modelling components by the authors contributes to the potential clinical translation of the model.

During *in silico* testing the authors managed to reproduce VA using *in vivo* experimental pacing patterns. However, clinical translation will require data acquisition from several subjects and further validation of all pacing schemes. Furthermore, whilst patients may undergo cardiac MRI examinations to evaluate post‐DOX reactive fibrosis, the invasive electrophysiology (EP) methodology used in this study would not be indicated in the absence of significant symptoms. Alternatively, it may be possible to assess the arrhythmogenic potential by extracting relevant EP data from common 12‐lead ECGs (Ly et al., 2025), in conjunction with cardiac late gadolinium enhancement cardiac MRI data. The limitations of this post‐gadolinium contrast cardiac MRI are low spatial resolution and low signal‐to‐noise ratio, decreasing the specificity for defining regions with arrhythmogenic potential. Alternative MRI parameters (e.g. T1 and T2 relaxation times or the myocardial perfusion reserve) may contribute to the classification of potential arrhythmogenic substrates.

Whilst swine heart disease models are successfully used experimentally to reproduce human cardiovascular disease and response to treatments, the model used in this study does not reflect optimally DOX toxicity observed in patients due to a relatively high level of myocardial fibrosis observed at only 9 weeks following exposure. However, after validation, this modelling approach may be generalizable to patients with other cardiomyopathies. The identification of patients most likely to benefit from implantable cardiac defibrillators (ICDs) not captured by ejection fraction thresholds is not yet established in guidelines, and significant gaps remain in risk stratification and patient selection for ICD therapy. Research into novel markers, including computational models that merge cardiac MRI and electrophysiology data, shows promise for personalised arrhythmia risk assessment but the clinical utility is not yet established. The next important step for successful clinical translation of the model by Villar‐Valero et al. (2026) will be to ‘open the method’ to clinicians and clinical scientists, that is by creating a user‐friendly software interface. Direct involvement of clinicians in the modelling tasks is then likely to contribute to further physiological validation, and will be essential for launching larger‐scale clinical studies and advancing computational modelling into clinical practice.

## Additional information

### Competing interests

None declared.

### Author contributions

R.C. and V.Z.: Conception or design of the work; Drafting the work or revising it critically for important intellectual content; Final approval of the version to be published; Agreement to be accountable for all aspects of the work. All authors have approved the final version of the manuscript and agree to be accountable for all aspects of the work. All persons designated as authors qualify for authorship, and all those who qualify for authorship are listed.

### Funding

None.

## Supporting information


Peer Review History
