# Supplementary material for: Cardiac digital twins: Modelling the arrhythmic substrate of chemotherapy
Source: J Physiol. 2026 Jan 29;604(13):5092–3. doi: 10.1113/JP290313 (PMC13327754; doi:10.1113/JP290313)
Supplement: Supplementary file 1 — Peer Review History [file TJP-604-5092-s001.pdf]

The Journal of Physiology

Cardiac Digital Twins: Modeling the Arrhythmic Substrate of Chemotherapy

Radomir Chabiniok and Vlad Zaha
DOI: 10.1113/JP290313

Corresponding author(s): Radomir Chabiniok (radomir.chabiniok@utsouthwestern.edu)

The referees have opted to remain anonymous.

Review Timeline:

Submission Date:	17-Nov-2025
Editorial Decision:	20-Nov-2025
Revision Received:	19-Dec-2025
Accepted:	03-Jan-2026

Senior Editor: Bjorn Knollmann

Reviewing Editor: Axel Loewe

Transaction Report:

(Note: With the exception of the correction of typographical or spelling errors that could be a source of ambiguity, letters and reports are not edited. Depending on transfer agreements, referee reports obtained elsewhere may or may not be included in this compilation. Referee reports are anonymous unless the Referee chooses to sign their reports.)

15-Dec-2025
We look forward to receiving your revised version soon!
Best wishes
Diana
JP

20-Nov-2025
Dear Dr Chabiniok,

Re: JP-TP-2025-290313 "**Cardiac Digital Twins: Modeling the Arrhythmic Substrate of Chemotherapy**" by Radomir Chabiniok and Vlad Zaha

Thank you for submitting your manuscript to The Journal of Physiology. It has been assessed by a Reviewing Editor and by 1 expert referee and we are pleased to tell you that it is acceptable for publication following minor revision.

Please advise your co-authors of this decision as soon as possible.

The review comments are copied at the end of this email.

Please address all the points raised and incorporate all requested revisions or explain in your Response to Referees why a change has not been made. We hope you will find the comments helpful and that you will be able to return your revised manuscript within 2 weeks. If you require longer than this, please contact journal staff: jp@physoc.org.

Your revised manuscript should be submitted online using the link in your Author Tasks: https://jp.msubmit.net/cgi-bin/main.plex

If you do not wish to submit a revised version of your manuscript, you must inform our journal staff (jp@physoc.org) or reply to this email to request withdrawal. Please note that a manuscript must be formally withdrawn from the peer review process at one journal before it may be submitted to another journal.

TRANSPARENT PEER REVIEW POLICY: To improve the transparency of its peer review process, The Journal of Physiology publishes online as supporting information the peer review history of all articles accepted for publication. Readers will have access to decision letters, including Editors' comments and referee reports, for each version of the manuscript, as well as any author responses to peer review comments. Referees can decide whether or not they wish to be named on the peer review history document.

LANGUAGE EDITING AND SUPPORT FOR PUBLICATION: If you would like help with English language editing, or other article preparation support, Wiley Editing Services offers expert help, including English Language Editing, as well as translation, manuscript formatting, and figure formatting at www.wileyauthors.com/eeo/preparation. You can also find resources for Preparing Your Article for general guidance about writing and preparing your manuscript at www.wileyauthors.com/eeo/prepresources.

REVISION CHECKLIST: Upload a full Response to Referees file. To create your 'Response to Referees' copy all the reports, including any comments from the Senior and Reviewing Editors, into a Microsoft Word, or similar, file and respond to each point, using font or background colour to distinguish comments and responses and upload as the required file type.

Please upload two versions of your manuscript text: one with all relevant changes highlighted and one clean version with no changes tracked. The manuscript file should include all tables and figure legends, but each figure/graph should be uploaded as separate, high-resolution files.

You may also upload:

- 'Potential Cover Art' for consideration as the issue's cover image
- Appropriate Supporting Information (video, audio or data set: see https://jp.msubmit.net/cgi-bin/main.plex

We look forward to receiving your revised submission.

If you have any queries, please reply to this email and we will be pleased to advise.

Yours sincerely,

Bjorn Knollmann
Senior Editor

EDITOR COMMENTS

Reviewing Editor:

Thank you for providing this perspectives article. The expert referee pointed out a number of minor points that should be addressed and 3 specific comments to further improve the manuscript that can be considered.

In addition, line 64: "in conjunction cardiac late" should probably read "in conjunction with cardiac late".

The reference list must be in alphabetical order, rather than numbered, to comply with our Journal format.

Please cite the focus paper (Villar-Valero et al) properly in the text, and in the References list.

Senior Editor:

I agree with the reviewing editor's recommendation.

REFEREE COMMENTS

Cardio-oncology is a newly formed discipline focused on studying cardiac complications (e.g. acute or chronic drug-induced cardiotoxicity) following oncologic therapies. Most studies have employed either clinical observations or preclinical/translational experimentation, with very few researchers exploring computational modelling tools that predict the heart electro-mechanical dysfunctions such as risk of arrhythmia or heart failure.

With this respect, in their article "Cardiac Digital Twins: Modeling the Arrhythmic Substrate of Chemotherapy", Drs Chabiniok and Zaha aimed to put in perspective the potential role of predictive digital twins in clinical cardio-oncology practice, crediting the initial and promising results recently obtained in the work published by Javier Villar-Valero and colleagues while also critically commenting on the current limitations of this particular work.

Overall, the perspective article is well written and capture a correct outlook regarding the contribution to physiology research and potential clinical translation of the results from Javier Villar-Valero and colleagues. The point of view will likely have a positive impact on the journal readers, with the hope that more researchers will start exploring and harness the capabilities of 3D image-based digital twins to dissect in detail and non-invasively some key physiological phenomena induced by chemotherapy regimes. These phenomena are always difficult to predict based solely on experimental and clinical observations in oncologic patients. Hence, future integration of open-source modelling tools into the routine clinical evaluation of chemotherapy effects could be of paramount help to the cardio-oncologists, which the authors have nicely emphasized.

Specific comments:

1) The authors could emphasize even more the important future role of digital twins used in cardio-oncology applications, especially to unveil subtle physiological consequences not easily observable in the clinics. It would be good to add a clinical opinion on the type of image-derived structural data needed to built accurate digital twins for long-term evaluation of arrhythmia risk (e.g. is MRI the best modality? what kind of clinical spatial resolution is adequate to characterize chemotherapy-induced fibrosis in oncologic patients).

2) In their modelling work, Javier Villar-Valero and colleagues included a supplementary material describing a basic comparison of their meshless LBM simulations with a well-known modelling benchmark as well as with the finite element approach. This should be briefly mentioned by Drs Chabiniok and Zaha in their perspective (since the main discussions is

about digital twins and modelling).

3) With respect to preclinical translational swine model(s): Drs Chabiniok and Zaha should note that swine models are widely used in research not only because the pig heart is similar in size with human heart (as they stated), but also because the pathophysiology (structure/function) in the setting of cardiovascular diseases as well as the response to treatment is highly reproducible and very similar to those humans.

Minor comments:

1) Usually the original article discussed in the perspective is cited not only in the article text but also in References (see publication here: https://pubmed.ncbi.nlm.nih.gov/41177993/ , and please ensure that all authors first/last names are correctly spelled out).

2) The word 'such' was overly used (even few times in the same sentence), please try to find a synonym.

3) Please check and correct the grammar, as there are a few mistakes/errors throughout the text.

END OF COMMENTS

Dear Editor,

We are pleased by the comments and are submitting a revised manuscript, addressing all the comments point-by-point herein.

EDITOR COMMENTS

Reviewing Editor:Reviewing Editor:

Thank you for providing this perspectives article. The expert referee pointed out a number of minor points that should be addressed and 3 specific comments to further improve the manuscript that can be considered. In addition, line 64: "in conjunction cardiac late" should probably read "in conjunction with cardiac late".

Corrected.

The reference list must be in alphabetical order, rather than numbered, to comply with our Journal format.

Please cite the focus paper (Villar-Valero et al) properly in the text, and in the References list.

The reference are sorted alphabetically, and the suggested paper is now cited as Villar-Valero et al. (2025).

Senior Editor:

I agree with the reviewing editor's recommendation.

REFEREE COMMENTS

Cardio-oncology is a newly formed discipline focused on studying cardiac complications (e.g. acute or chronic drug-induced cardiotoxicity) following oncologic therapies. Most studies have employed either clinical observations or preclinical/translational experimentation, with very few researchers exploring computational modelling tools that predict the heart electro-mechanical dysfunctions such as risk of arrhythmia or heart failure. With this respect, in their article "Cardiac Digital Twins: Modeling the Arrhythmic Substrate of Chemotherapy", Drs Chabiniok and Zaha aimed to put in perspective the potential role of predictive digital twins in clinical cardio-oncology practice, crediting the initial and promising results recently obtained in the work published by Javier Villar-Valero and colleagues while also critically commenting on the current limitations of this particular work.

Overall, the perspective article is well written and capture a correct outlook regarding the contribution to physiology research and potential clinical translation of the results from Javier Villar-Valero and colleagues. The point of view will likely have a positive impact on the journal readers, with the hope that more researchers will start exploring and harness the capabilities of 3D image-based digital twins to dissect in detail and non-invasively some key physiological phenomena induced by chemotherapy regimes. These phenomena are always difficult to predict based solely on experimental and clinical observations in oncologic patients. Hence, future integration of open-source modelling tools into the routine clinical evaluation of chemotherapy effects could be of paramount help to the cardio-oncologists, which the authors have nicely emphasized.

Specific comments:

1) The authors could emphasize even more the important future role of digital twins used in cardio-oncology applications, especially to unveil subtle physiological consequences not easily observable in the clinics. It would be good to add a clinical opinion on the type of image-derived structural data needed to built accurate digital twins for long-term evaluation of arrhythmia risk (e.g. is MRI the best modality? what kind of clinical spatial resolution is adequate to characterize chemotherapy-induced fibrosis in oncologic patients).

We have addressed this comment including the following text in the manuscript:

“Echocardiography is the most prevalent cardiac imaging modality. However, limited acoustic windows and variability in technical acquisition performance decrease the sensitivity of this method especially for early mechanical changes. In contrast, the access to cardiac magnetic resonance imaging (MRI) is more limited than to echocardiography, but cardiac MRI is the gold standard for measurement of cardiac morphology and mechanical function, providing measurements with a high level of accuracy. Nevertheless, even the best current clinical methods taken in isolation have a limited predictive ability for negative outcomes (Lyon et al., 2022).”

2) In their modelling work, Javier Villar-Valero and colleagues included a supplementary material describing a basic comparison of their meshless LBM simulations with a well-known modelling benchmark as well as with the finite element approach. This should be briefly mentioned by Drs Chabiniok and Zaha in their perspective (since the main discussions is about digital twins and modelling).

We addressed this comment by including the text:

“The computations therefore do not need to be outsourced from the clinical department. Javier Villar-Valero and colleagues validated their LBM implementation against the well-known modeling benchmark by Niederer et al. (2011): they demonstrated an excellent

agreement in the wavefront propagation and revealed a consistent 140-fold speedup in computational time compared to FEM."

3) With respect to preclinical translational swine model(s): Drs Chabiniok and Zaha should note that swine models are widely used in research not only because the pig heart is similar in size with human heart (as they stated), but also because the pathophysiology (structure/function) in the setting of cardiovascular diseases as well as the response to treatment is highly reproducible and very similar to those humans.

We addressed this point by updating the text, highlighting the limitation of the cardiotoxicity model used in this study:

"While swine heart disease models are successfully used experimentally to reproduce human cardiovascular disease and response to treatments, the model used in this study has does not reflect optimally DOX toxicity observed in patients due to a relatively high level of myocardial fibrosis observed at only 9 weeks post-exposure."

Minor comments:

1) Usually the original article discussed in the perspective is cited not only in the article text but also in References (see publication here:

https://pubmed.ncbi.nlm.nih.gov/41177993/ , and please ensure that all authors first/last names are correctly spelled out).

We are now citing the article. We have copy-pasted the authors first/last names from the pubmed and assuming they are correct there.

2) The word 'such' was overly used (even few times in the same sentence), please try to find a synonym.

During the revision, we modified the text and kept the word "such" only in the opening sentence. Specifically: "*Anthracyclines, such as doxorubicine (DOX), …."*

3) Please check and correct the grammar, as there are a few mistakes/errors throughout the text.

We corrected the grammar and typos.

Dear Dr Chabiniok,

Re: JP-TP-2025-290313R1 "**Cardiac Digital Twins: Modeling the Arrhythmic Substrate of Chemotherapy**" by Radomir Chabiniok and Vlad Zaha

We are pleased to tell you that your paper has been accepted for publication in The Journal of Physiology.

TRANSPARENT PEER REVIEW POLICY: To improve the transparency of its peer review process, The Journal of Physiology publishes online as supporting information the peer review history of all articles accepted for publication. Readers will have access to decision letters, including Editors' comments and referee reports, for each version of the manuscript, as well as any author responses to peer review comments. Referees can decide whether or not they wish to be named on the peer review history document.

The last Word (or similar) version of the manuscript provided will be used by the Production Editor to prepare your proof. When this is ready you will receive an email containing a link to Wiley's Online Proofing System. The proof should be thoroughly checked and corrected as promptly as possible.

Authors should note that it is too late at this point to offer corrections prior to proofing. Major corrections at proof stage, such as changes to figures, will be referred to the Editors for approval before they can be incorporated. Only minor changes, such as to style and consistency, should be made at proof stage. Changes that need to be made after proof stage will usually require a formal correction notice.

All queries at proof stage should be sent to: TJP@wiley.com.

Please note that Translational Perspective articles are not typically covered by institutional open access agreements with our publisher, Wiley. Wiley do not offer article processing charge (APC) discounts for smaller article types in hybrid subscription journals, meaning that if you wish for your Perspective to be published Open Access, you will have to pay the full APC. As such, we recommend authors publish Translational Perspectives 'behind the paywall', where they will become freely accessible after a 12-month embargo (i.e. please select the NON open access option via Wiley Author services during proofing).

Should you wish to pay for Open Access, you will be able to place an order by logging into Wiley Author services.

• You can check if your funder or institution has a Wiley Open Access Account here: https://authorservices.wiley.com/author-resources/Journal-Authors/licensing-and-open-access/open-access/author-compliance-tool.html.

Yours sincerely,

Bjorn Knollmann
Senior Editor
The Journal of Physiology

P.S. - You can help your research get the attention it deserves! Check out Wiley's free Promotion Guide for best-practice recommendations for promoting your work at www.wileyauthors.com/eeo/guide. You can learn more about Wiley Editing Services which offers professional video, design, and writing services to create shareable video abstracts, infographics, conference posters, lay summaries, and research news stories for your research at www.wileyauthors.com/eeo/promotion.

IMPORTANT POINTS TO NOTE FOLLOWING ACCEPTANCE OF YOUR PAPER:

• IMPORTANT NOTICE ABOUT OPEN ACCESS: To assist authors whose funding agencies mandate public access to published research findings sooner than 12 months after publication, The Journal of Physiology allows authors to pay an Open Access (OA) fee to have their papers made freely available immediately on publication.

The Corresponding Author will receive an email from Wiley with details on how to register or log-in to Wiley Authors Services where you will be able to place an order

• You can check if your funder or institution has a Wiley Open Access Account here: https://authorservices.wiley.com/author-resources/Journal-Authors/licensing-and-open-access/open-access/author-compliance-tool.html.

• TRANSPARENT PEER REVIEW POLICY: To improve the transparency of its peer review process, The Journal of

Physiology publishes online as supporting information the peer review history of all articles accepted for publication. Readers will have access to decision letters, including Editors' comments and referee reports, for each version of the manuscript as well as any author responses to peer review comments. Referees can decide whether or not they wish to be named on the peer review history document.

• You can help your research get the attention it deserves! Check out Wiley's free Promotion Guide for best-practice recommendations for promoting your work at: www.wileyauthors.com/eeo/guide. You can learn more about Wiley Editing Services which offers professional video, design, and writing services to create shareable video abstracts, infographics, conference posters, lay summaries, and research news stories for your research at: www.wileyauthors.com/eeo/promotion.

• If you would like to receive our 'Research Roundup', a monthly newsletter highlighting the cutting-edge research published in The Physiological Society's family of journals (The Journal of Physiology, Experimental Physiology, Physiological Reports, The Journal of Nutritional Physiology, and The Journal of Precision Medicine: Health and Disease), please click this link, fill in your name and email address and select 'Research Roundup':
https://www.physoc.org/journals-and-media/membernews

EDITOR COMMENTS

Reviewing Editor:

Thank you for addressing the issues and improving this perspectives article further.

Senior Editor:

The manuscript is now acceptable for publication. Thank you for your excellent contribution to the Journal!

END OF COMMENTS
